# Cultural Safety within the Indigenous Health Context: Findings from a Review of Reviews

**DOI:** 10.1093/geroni/igab046.3148

**Published:** 2021-12-17

**Authors:** Juanita-Dawne Bacsu, Christina Chakanyuka, Andrea DesRoches, Jennifer Walker, Jessy Dame, Megan O'Connell, Paisly Symenuk, Lisa Bourque Bearskin

**Affiliations:** 1 University of Saskatchewan, Saskatoon, Saskatchewan, Canada; 2 University of Victoria, Victoria, British Columbia, Canada; 3 University of Saskatchewan, University of Saskatchewan, Saskatchewan, Canada; 4 Laurentian University, Sudbury, Ontario, Canada; 5 UBC, Vancouver, British Columbia, Canada; 6 Independent, Edmonton, Alberta, Canada; 7 Thompson Rivers University, Kamloops, British Columbia, Canada

## Abstract

First Nations, Inuit, and Métis older adults often face systemic barriers to accessing culturally safe and equitable healthcare, including racism, structural injustice, and a historical legacy of colonialism. However, there is a paucity of knowledge on cultural safety interventions and implementation strategies in care for older adults. This presentation aims to: 1) explore persistent barriers to achieving health equity and advancing cultural safety in healthcare; and 2) identify cultural safety interventions to improve healthcare for Indigenous older adults. Guided by Arksey and O’Malley’s scoping review framework, we conducted a review of reviews published between January 2010 to December 2020 on Indigenous cultural safety in healthcare. We searched five databases (CINAHL, PubMed, Scopus, Web of Science, and Google Scholar) and hand-searched reference lists of relevant articles. We conducted a thematic analysis to identify patterns and themes in the literature. Key barriers to achieving health equity and advancing cultural safety in healthcare included care providers lacking knowledge of Indigenous culture, power imbalances, racism, and discrimination. A range of cultural safety interventions were identified, from education and training initiatives for healthcare providers (emergency physicians and occupational therapists) to collaborative partnerships with First Nations, Inuit, and Métis communities. As First Nations, Inuit, and Métis populations age, there is a growing need for safe healthcare services for Indigenous older adults, and these findings suggest focusing on healthcare providers knowledge and attitudes is key. Research is necessary to develop, implement, and evaluate cultural safety interventions aimed at healthcare providers to improve healthcare for Indigenous older adults.

